# Comparative Metabolomic and Lipidomic Analysis of Phenotype Stratified Prostate Cells

**DOI:** 10.1371/journal.pone.0134206

**Published:** 2015-08-05

**Authors:** Tanya C. Burch, Giorgis Isaac, Christiana L. Booher, Johng S. Rhim, Paul Rainville, James Langridge, Andrew Baker, Julius O. Nyalwidhe

**Affiliations:** 1 Department of Microbiology and Molecular Cell Biology, Eastern Virginia Medical School, Norfolk, Virginia, United States of America; 2 Leroy T. Canoles Jr. Cancer Research Center, Eastern Virginia Medical School, Norfolk, Virginia, United States of America; 3 Waters Corporation, Milford, Massachusetts, United States of America; 4 Center for Prostate Disease Research, Uniformed Services University of the Health Sciences, Bethesda, Maryland, United States of America; 5 Waters Corporation, Manchester, United Kingdom; Hormel Institute, University of Minnesota, UNITED STATES

## Abstract

Prostate cancer (PCa) is the most prevalent cancer amongst men and the second most common cause of cancer related-deaths in the USA. Prostate cancer is a heterogeneous disease ranging from indolent asymptomatic cases to very aggressive life threatening forms. The goal of this study was to identify differentially expressed metabolites and lipids in prostate cells with different tumorigenic phenotypes. We have used mass spectrometry metabolomic profiling, lipidomic profiling, bioinformatic and statistical methods to identify, quantify and characterize differentially regulated molecules in five prostate derived cell lines. We have identified potentially interesting species of different lipid subclasses including phosphatidylcholines (PCs), phosphatidylethanolamines (PEs), glycerophosphoinositols (PIs) and other metabolites that are significantly upregulated in prostate cancer cells derived from distant metastatic sites. Transcriptomic and biochemical analysis of key enzymes that are involved in lipid metabolism demonstrate the significant upregulation of choline kinase alpha in the metastatic cells compared to the non-malignant and non-metastatic cells. This suggests that different *de novo* lipogenesis and other specific signal transduction pathways are activated in aggressive metastatic cells as compared to normal and non-metastatic cells.

## Introduction

In 2015, it is estimated that there will be 220,800 new prostate cancer (PCa) cases and 27,540 deaths due to the disease in the USA [1]. This makes PCa the most prevalent cancer amongst men and the second most common cause of cancer related-deaths in the country. Although PCa has a long latent period of development, clinically, the disease has very heterogeneous phenotypes ranging from indolent asymptomatic cases to very aggressive life threatening and lethal forms. One of the most critical challenges in the management of PCa is to distinguish patients with indolent asymptomatic disease from those with very aggressive forms who would benefit from definitive treatment. Many new prostate cancer biomarkers have recently emerged, but only a few have shown significant clinical value [2–7]. Currently, it is not possible to distinguish indolent from aggressive forms of prostate cancer. This inability to accurately predict the aggressiveness of PCa based solely on standard clinicopathologic features underscores the need to explore the ability of novel biomarkers to enhance outcome prediction at biopsy and to understand the molecular basis of PCa metastasis. Therefore, additional biomarkers with high sensitivity and specificity, and preferably obtained minimal invasiveness are urgently needed for PCa diagnosis and prognosis.

Potential biomarkers for progression of PCa from the precursor lesion to organ confined primary tumor and finally to distant metastasis may include genes, proteins and metabolites. Metabolites are the end products of molecular pathways that are initiated at genomic, transcriptomic, and proteomic levels. These metabolites may serve as surrogates for disease stratification and potentially as useful prognostic and diagnostic biomarkers. Metabolomics of prostate cancer is currently being studied to screen for biomarkers with high sensitivity and specificity [8–11]. However, to date no comparative metabolomic analyses of disease stratified prostate cancer cell lines has been performed. Here, we provide comparative metabolomics and lipidomics profiling data from 5 prostate cancer cells obtained from patients with different disease phenotypes. This study reveals a trend in the expression profiles of specific classes of lipids and metabolites in cell lines with different tumorigenic phenotypes. Some of these molecules may be potentially involved in the modulation of physiological and metabolic processes that are associated with prostate cancer disease progression and the promotion of the metastatic phenotype.

## Materials and Methods

### Prostate Cell Lines and Cultures

The following prostate derived cells were used for metabolomic analyses. RWPE-1 cells (CRL-11609) were obtained from American Type Culture Collection (ATCC (Manassas, VA). These cells are non-neoplastic adult human prostatic epithelial cells from a Caucasian male donor that were immortalized with human papillomavirus 18 as previously described [12]. LNCAP (CRL-1740) cells were also obtained from ATCC. These prostatic cells were originally derived from the left supraclavicular lymph node metastatic site from a Caucasian male donor and are tumorigenic in nude mice [13]. The RC77N-E and RC77T-E cells were a kind gift from Dr. Johng S. Rhim [14–15]. These cells were derived from an African American prostate cancer patient and have been immortalized with HPV-16E6E7 [14–15]. The RC77T-E cells were derived from malignant adenocarcinoma tissue, whereas the RC77N-E cells were obtained from non-malignant tissue from the same prostate. The RC77T-E cells produced tumors in SCID mice whereas the RC77N-E cells produced no tumor in SCID mice [14–15]. MDAPCa2b (CRL-2422) cells were also obtained from ATCC. These prostatic cells were originally derived from a bone metastatic site from an African American male donor. These cells produce tumors in nude mice when injected either subcutaneously or orthotopically (intraprostatic) [16]. All the five cell lines possess the androgen receptor and are responsive to androgen stimulation. The RWPE-1, RC77N-E, RC77T-E cells were grown in KSFM medium (Life-Technologies); LNCAP in cells in RPMI (Life-Technologies) and MDAPCa2b cells in HPC1 medium AthenaES). All the media were supplemented with 5% fetal bovine serum and the cells were grown at 37°C in humidified air with 5% CO_2_ as has been previously described [17]. Choline kinase α rabbit monoclonal antibody (D5X9W) was from Cell Signaling Technology (Beverly, MA). GAPDH rabbit polyclonal antibody (sc-25778) was from Santa Cruz Biotechnology (Santa Cruz, CA).

### Sample Preparation and Metabolite Extraction

The five prostate derived cells were cultured to 80% confluence and the adherent cells detached using 5 mM EDTA in PBS from three independent experiments. The harvested cells were washed three times with phosphate buffered saline (PBS) to remove any traces of culture medium. The cells were resuspended in distilled water before lysis and homogenization by 3 cycles of freezing and thawing in liquid nitrogen. The samples were sonicated for 20 seconds three times before centrifugation to remove cell debris and unbroken cells. The supernatant was collected and the protein concentration was determined by BCA assay. Total cell lysates with normalized protein concentrations (100 μg) were homogenized in 1 mL of water and mixed with 3.75 mL of a mixture of chloroform/methanol (1:2 v/v ratio). The mixture was vortexed for 10–15 min before adding 1.25 mL of chloroform and vortexing for 1 min. 1.25 mL of water were added and the mixture vortexed for another 1 min. The samples were centrifuged at 13000 x g for 10 minutes. The upper phase containing water soluble metabolites was collected for polar metabolite analysis. The protein disc interface was carefully pierced with a pipette tip to collect the lower organic phase containing hydrophobic metabolites. The organic phase and water layer were collected in separate siliconized tubes and evaporated to dryness in a SpeedVac at room temperature. The metabolite samples were reconstituted in the appropriate liquid chromatography buffer A prior to MS analysis. For lipidomics analysis, samples were reconstituted in 200 μl 1:1 chloroform/methanol buffer and the hydrophilic metabolite samples were reconstituted in 5% acetonitrile/water.

### Mass Spectrometry Metabolomic and Lipidomic Profiling

UPLC was performed using an ACQUITY *i*-Class system (Waters, Milford, MA, USA) coupled to a SYNAPT G2-S mass spectrometer (Waters, Milford, MA). The running conditions were as follows: 2 μl sample injections of the metabolites were used in 20 minute UPLC runs. For the lipid separation an ACQUITY UPLC CSH C18 (2.1 x 100 mm, 1.7μm) column was used with a column temperature of 55°C and at a flow rate of 400 μl/min. The mobile phase buffer A was acetonitrile/water (60/40) with 10 mM ammonium formate and 0.1% formic acid. Mobile phase buffer B was isopropanol/acetonitrile (90:10) with 10 mM ammonium formate and 0.1% formic acid. The initial LC gradient conditions were 40% buffer B rising to 70% in 12 minutes to a maximum 99% after 6 min before re-equilibration for 2 minutes at 40% B. The SYNAPT G2-HDMS mass spectrometer was used for MS acquisition under the following conditions: Acquisition mode, MS^E^ operated at 25,000 resolution; ionization mode; electrospray ionization both positive and negative mode; capillary voltage, 2.0 KV (positive mode) and 1.0 KV (negative mode); cone voltage, 30V; desolvation temperature, 550°C; desolvation gas, 900 L/Hr; source temperature 120°C and an acquisition range of 100 to 2000 m/z. with mobility experiments performed with nitrogen and using Leu Enkephalin lock mass. The MS conditions for the analysis of the polar metabolites were essentially as described for the lipids with the following chromatographic conditions: ACQUITY UPL BEH amide (2.1 x 150 mm, 1.7μm) with a column temperature of 45°C. The flow rate was 400 μl/min with; mobile phase A, 95% acetonitrile/ ammonium bicarbonate 10 mM (pH9) 5% in the negative mode for basic conditions. For acidic conditions the mobile phases were changed to 100% acetonitrile (mobile phase A) and 100% water (mobile phase B) both containing 0.1% formic acid for both positive and negative acquisition modes.

### RT-PCR Analysis and Western Blot Analysis

Total RNA was extracted from the different cells using (RNeasy; Qiagen), and cDNA was synthesized with SuperScript II reverse transcriptase (Invitrogen). To check for conserved transcripts for ChoK-α, LPCAT1, LPCAT2, and LPCAT3, specific primers were designed to generate comparable PCR products with approximately the same number of base pairs and identical sizes using NCBI Primer-BLAST (http://blast.ncbi.nlm.nih.gov/Blast.cgi). For quantitative real-time RT-PCR, 1μg of total RNA was used for cDNA synthesis with a reverse transcription kit (Qiagen). GAPDH was used as endogenous control for normalization. The primers used for quantitative real time RT-PCR are listed in S1 Table. For each of the genes, PCR reactions were performed in duplicate using the SYBR green assay on a Bio-Rad CFX-96 system. Quantitation was done using ΔΔCt method as per manufacturer's instructions (www.sabiosciences.com). GAPDH a housekeeping gene was used for normalization and calculation of fold changes between the expression profiles of the genes in the different cell lines. The relative expression of *ChoK-α*, *LCAT1*, *LPCAT2* and *LPCAT3* between the cell lines were performed using the normal epithelial RWPE-1 cell line as control. A significance threshold of ≥ 2.0 was used as the cut-off for upregulation. The real time RT-PCR results were validated to confirm the expression of ChoK-α and GAPDH protein in the 5 cell lines using Immunoblots as we have previously described [17].

## Results

**Fig 1:**
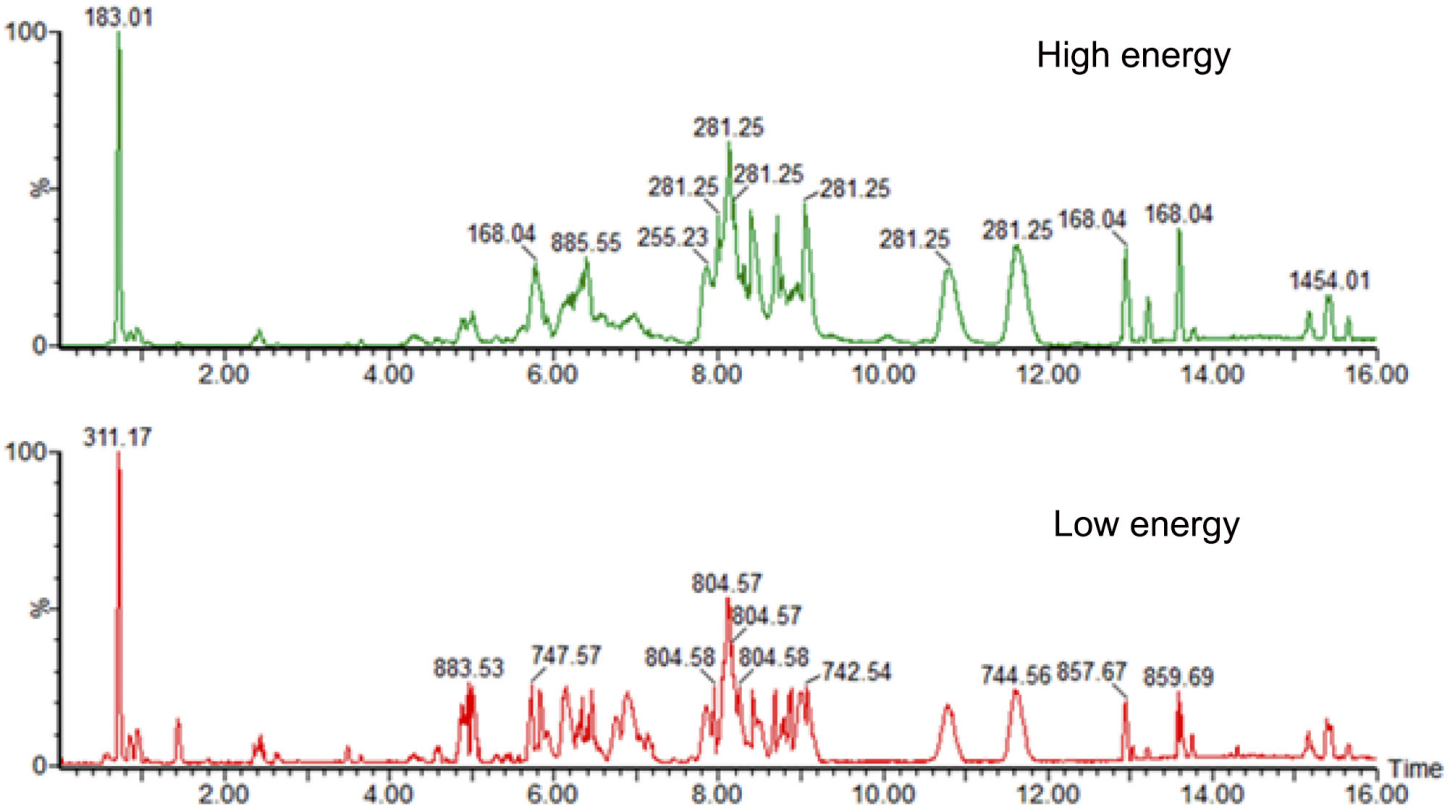
Analysis of lipids extracted from prostate cancer cell lines using UPLC/MS-MS. (A). A chromatogram acquired in high-energy mode. (B). A chromatogram acquired in low energy mode within the same experiment.

A novel UPLC/MS^E^ data-independent method that provides molecular and structural information from every detectable component in a liquid chromatography separation was used to ensure maximizing data quality and coverage. This method provides informative mass spectrometry data that includes precursor exact ion mass in low energy and corresponding fragment ion spectra in high energy. This semi-quantitative approach requires little prior knowledge of the sample and is unbiased and reproducible. The results from comparing MS^E^ with targeted MS/MS analysis have shown that the same metabolite structure was assigned in 95.7% of the cases [18]. Fig 1 shows the chromatogram for the separation of various lipid classes in the pooled sample by electrospray ionization in the negative ion mode. Both low and high energy data was acquired within the same run (MS^E^) and the data from the two experiments were subsequently aligned for structural elucidation. The analysis of the acquired data was accomplished using the novel Progenesis QI v1.0 (Nonlinear Dynamics, Newcastle, UK) data processing and statistical tool. Progenesis QI allows for the performance of differential analysis of high resolution UPLC-MS metabolomics data across different biological samples. This strategy allows for the identification and quantitation of potential biomarkers. Key to this data processing and analysis is the ability of Progenesis QI to distinguish biological variation and metabolic changes from analytical interferences. It is crucial that each sample is randomized and with a minimum of three technical replicates to ensure for statistical validity of the data.

For this study, the biological groups were randomized and injected three times with a set of QC pooled sample runs. The workflow starts with UPLC-MS raw data file importing. Details of data file format and a list of expected adducts are entered to facilitate the handling of data import followed by automatic retention time alignment. The imported data is presented as a low and high energy ion intensity map of m/z versus retention time enabling the visual check of the chromatography. Metabolomics experiments involve large a amount of sample runs that may result in a shift in retention time. The LC/MS data was first aligned to correct any retention time drift between analytical runs. After retention time alignment, automatic peak detection, normalization, deconvolution, compound quantitation, identification and statistical analysis was performed. Alignment of the data runs allows a common pattern of compound ion detection to be performed across all the runs in the experiment. Adducts of the same compound are automatically grouped during deconvolution based on their retention time and mass difference. The deconvolution process uses the list of defined adducts provided when importing the raw data. Table 1 summarizes the age, ethnicity, morphology, clinical and pathological features of the patients from whom these cell lines were obtained.

**Table 1:** Clinical features of the patients from whom prostate cell lines were derived.

Cell Line	Age	Race	Morphology/ AR & Response	Clinical Stage	Tumor Grade	Gleason Score
RWPE-1	54	CA	Epithelial (AR+)	Non-malignant	N/A	N/A
LNCAP	50	CA	Epithelial (AR+)	Adenocarcinoma metastatic Lymph node tumor	N/A	N/A
RC77N-E	62	AA	Epithelial (AR+)	Non-malignant	N/A^c^	N/A^c^
RC77T-E	62	AA	Epithelial (AR+)	Primary Adenocarcinoma	Poorly differentiated	7
MDA PCa 2b	63	AA	Epithelial (AR+)	Adenocarcinoma metastatic bone tumor	N/A	N/A

### Identification of Metabolites and Lipids in Prostate Cell Lines

**Fig 2:**
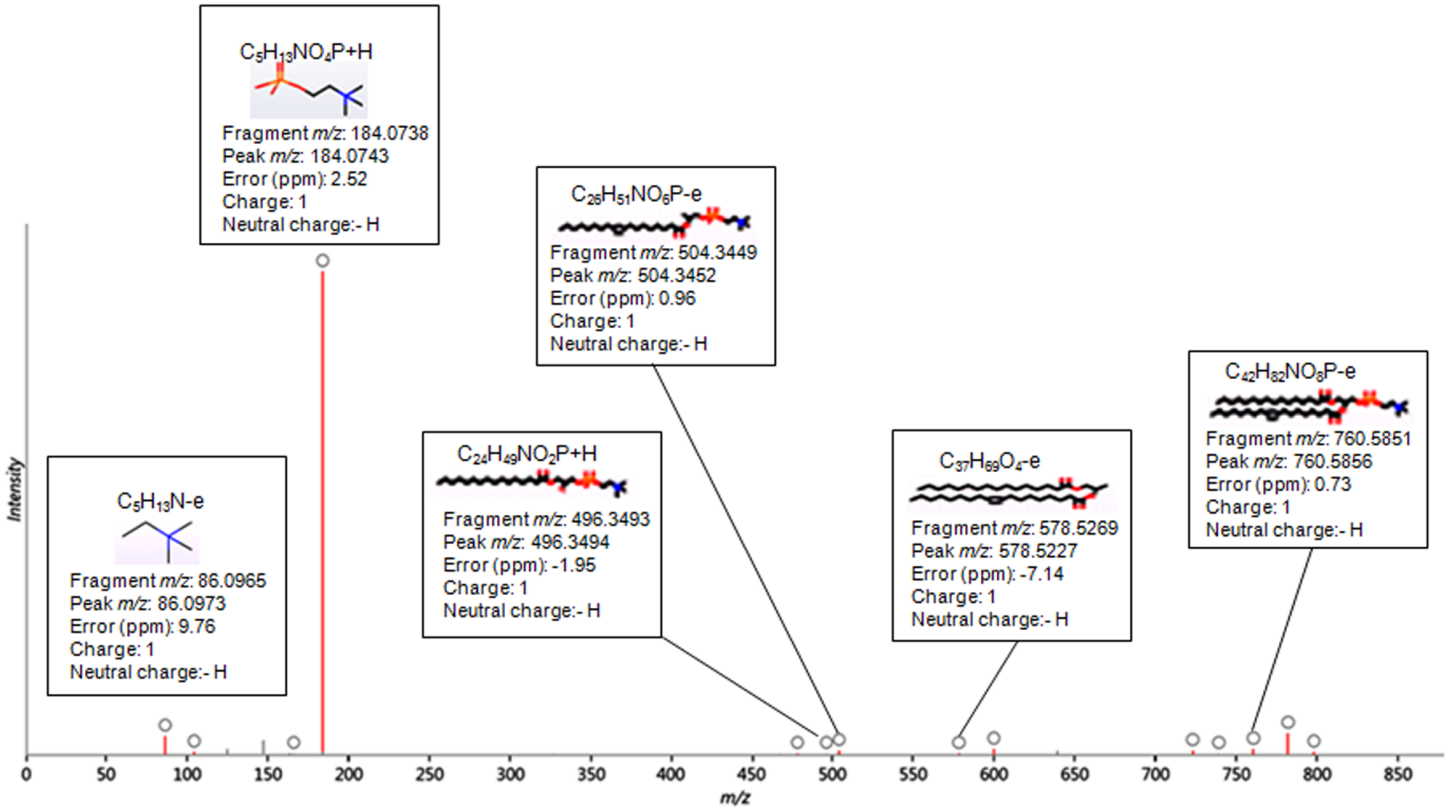
Tandem mass spectrometry spectrum. Fragmentation trace for compound 8.13_759.5775n in the positive ion mode with the identification of PC (16:0/18:1(9Z)) (1-hexadecanoyl-2-(9Z-octadecenoyl)-sn-glycero-3-phosphocholine). The measured spectra which are matched against the in silico fragment ions are highlighted in red. The circle on the top of the red spectra shows the structure, measured m/z, theoretical m/z and corresponding mass error in ppm.

The identification of the metabolites was based on exact mass precursor ion, theoretical isotopic distribution, retention time and high energy fragment ion information. To improve the confidence in the compound identification theoretical, fragmentation of a candidate list of compounds was performed and then matched to the resulting *‘in silico’* fragmentation against the measured fragments for a compound. The candidate molecules were selected from compound databases (LipidMAPS database for lipids and Human Metabolome Database (HMDB) for polar metabolites) based on the exact mass within a specified error range given in parts per million (ppm). Using this list of candidates, the fragmentation algorithm generates all possible fragments for a candidate compound in order to match the fragment mass with the measured peaks in the experimental data. The measured spectra are then matched against the in silico fragment ions. The different lipid species of PC, PE, PS, PI and TG are listed with the two fatty acyl groups separated with a slash, e.g. PC 16:0/18:1. Fig 2 shows a representative fragmentation trace for compound 8.13_759.5775n in the positive mode lipidomics experiments with the identification of PC(16:0/18:1) (1-hexadecanoyl-2-(octadecenoyl)-sn-glycero-3-phosphocholine). The measured spectra which are matched against the in silico fragment ions are highlighted in red. The circle on the top of the red spectra shows the structure, measured m/z, theoretical m/z and corresponding mass error in ppm. In this approach, lipid species are identified at level of head group plus total acyl carbons: total double bonds. The detected intensities, each defined by an intact ion mass/charge (m/z) and a characteristic fragment m/z, are herein described as ‘‘apparent lipid molecular species”.

### Metabolite and Lipid profiles of Prostate Cell Lines

**Fig 3:**
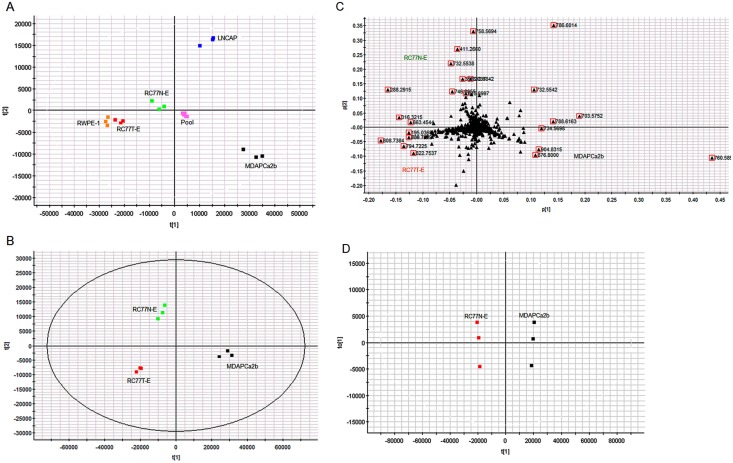
Comparative analysis of metabolite profiles of the cell lines. (A) PCA scores plot of the metabolite profiles of the five prostate cell lines, RWPE-1, LNCAP, RC77N-E, RC77T-E, MDAPCa2b and pooled sample. (B) PCA scores plot of the metabolites of the three African American cell lines RC77N-E, RC77T-E and MDAPCa2b. (C) Corresponding loadings plot from PCA analysis of the three cell lines. (D) Corresponding OPLS-DA analysis of the two African American cell lines RC77N-E and MDAPCa2b.

The data from Progenesis QI analysis was exported to EZinfo for detailed multivariate statistical analysis such as Principal component analysis (PCA), orthogonal partial least squares—discriminant analysis (OPLS-DA) and S-plot. Using these multivariate statistical tools, regardless the complexity of the sample, it is easier to determine the features that change between the different cell lines for further identification and targeted analysis. PCA was performed for the acquired metabolomic data to identify whether the metabolite profiles of the different prostate cell lines could be used to differentiate tumorigenic phenotypes. The scores and loadings plot for the PCA model of the five different cell lines and the pooled samples for lipid metabolites in the positive mode is shown in Fig 3a. The clustering of the pooled samples at the origin of the PCA plot indicates the robustness of the analysis. Different comparative PCA analyses were performed amongst the 5 cell lines. As an example, the scores and loadings plot for the PCA model of the three cell lines RC77N-E, RC77T-E and MDAPCa2b with different tumorigenic phenotypes is shown in Fig 3b. Three groups were separated on the basis of the PCA analysis, reflecting the tumorigenic features of these cell lines. The loadings plot indicates the exact mass retention time pairs (EMRT’s) that contributed towards the groupings in the scores plot, Fig 3c. The major EMRT’s that contribute significantly in differentiating the three cell lines have m/z 760.5855, 876.800, 904.8315 (upregulated in MDAPCa2b), 758.5694, 411.2660, 732.5538 (upregulated in RC77N-E) and 808.7384, 794.7225, 822.7537 (upregulated in RC77T-E).

**Fig 4:**
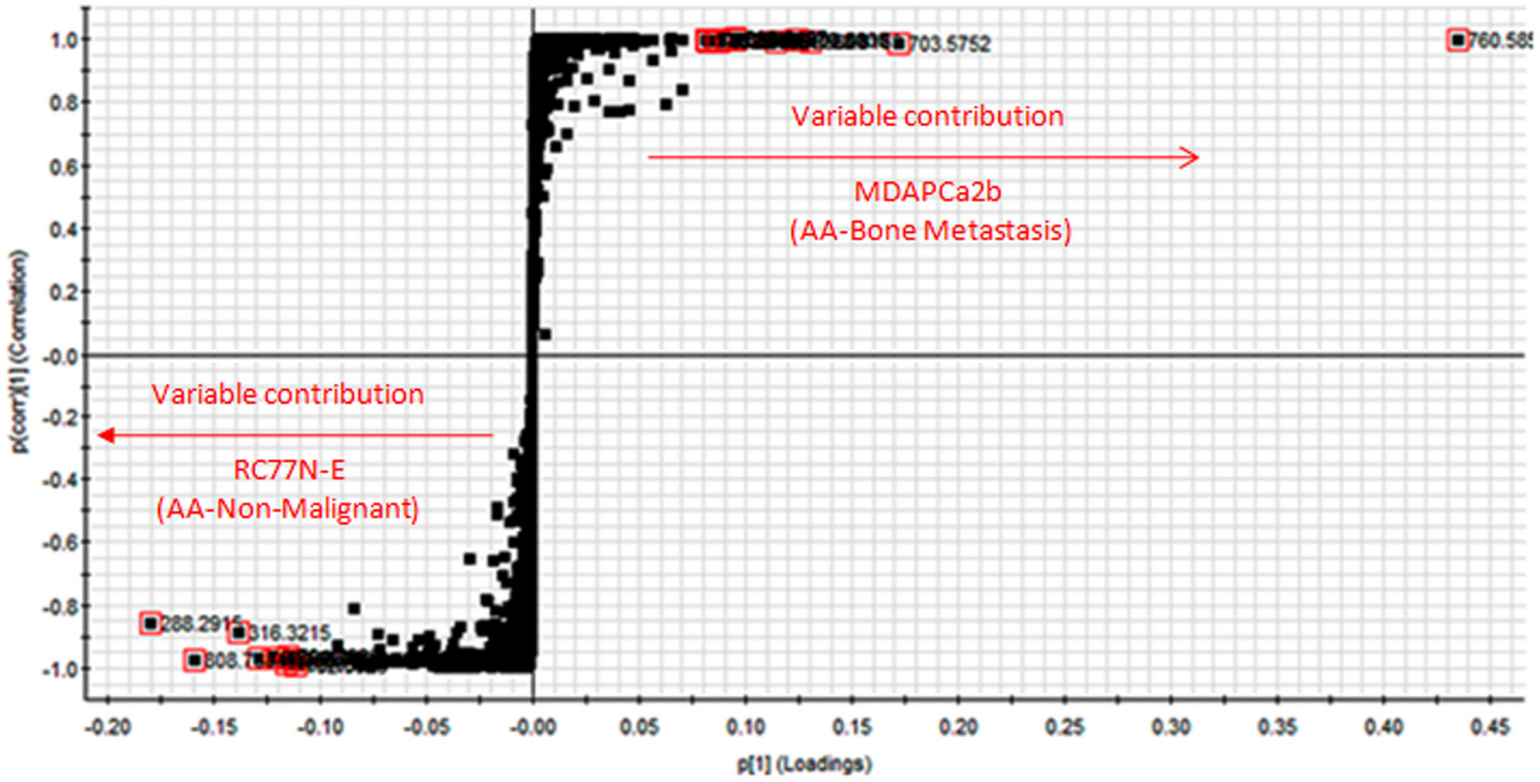
Orthogonal partial least square discriminant analysis. S-plot for the OPLS-DA data for the two African American cell lines RC77N-E and MDAPCa2b. The axis represents the reliability and magnitude of each EMRT.

**Fig 5:**
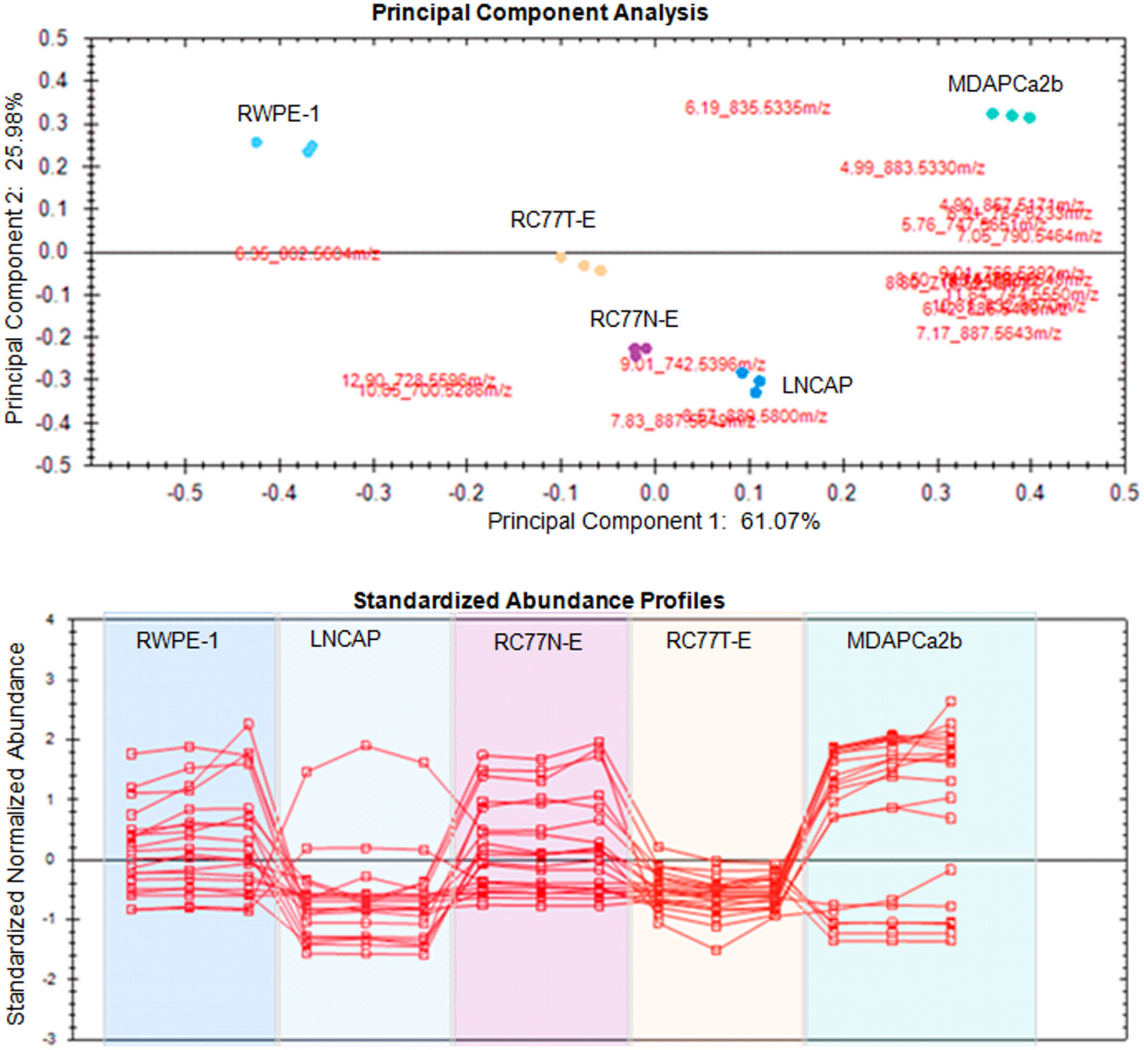
Principal component and trend analyses. (A) Principal component analysis and (B) Trend Plots for metabolites that are upregulated in MDAPCa2b vs RC77N-E and RC77T-E.

In an attempt to identify metabolic markers that differentiate most between the non-malignant, RC77N-E and the bone metastatic MDAPCa2b, a binary comparison was performed using Orthogonal partial least square discriminant analysis (OPLS-DA) modeling. The scores plot of the OPLS-DA data further separated the RC77N-E and MDAPCa2b cell lines (Fig 3d). The OPLS-DA data were visualized using an S-plot (Fig 4). The X-axis in the S-plot represents the reliability and magnitude of each EMRT to the group difference and the Y-axis represents the confidence of each EMRT contribution to the group difference. Similar binary comparison can be performed between the different cell lines to identify the EMRT’s that contribute the most for the group separation. The EMRT’s with the greatest magnitude and reliability for the RC77N-E and MDAPCa2b cell lines were identified as PC(16:0/18:1) (m/z 760.5855, ESI+), and PC(16:0/16:1) (m/z 731.5459, ESI+). The other most significantly upregulated compound with the highest fold increase has a neutral mass of 1519.1517. The MS/MS fragmentation data is not sufficient for the unambiguous identification of the compound. Some of the compounds that contribute to the separation of these cell lines are summarized in Table 2. Subsequent trend analysis of metabolite expression profiles amongst the three RC77N-E, RC77T-E and MDAPCa2b cell lines reveal a group of metabolites that are significantly upregulated in the bone metastatic MDAPCa2b cells compared to the non-malignant RC77N-E and the primary adenocarcinoma RC77T-E cell lines (data not shown). These metabolites are potential markers of prostate cancer metastasis. Fig 5 shows the PCA analysis and trend plots for these molecules amongst the 5 cell lines. Table 2 summarizes the identifications, molecular properties and statistical analysis parameters for the metabolites that are differentially expressed between the non-malignant RC77N-E and the bone metastatic MDAPCa2b cell lines. The identifications are for lipids and polar metabolites in both negative and positive acquisition modes. S1 Fig shows representative low- and high-energy spectra for the identification of a differentially expressed lipid between RC77N-E and MDAPCa2b. Similar analyses have been performed on the same samples using MS data acquired in the negative mode and the results of the lipid metabolites amongst the 5 cell lines are summarized in Tables 3 and 4.

**Table 2:** Significantly upregulated lipid molecular species between the bone metastatic MDAPCa2b cells and the non-malignant RC77N-E cells.

Compound LC MS/MS ID	LMID	Common Name	Exact Neutral Monoisotopic Mass	Maximum Fold increase	Highest Mean Cell Line	Lowest Mean Cell Line	P value
16.5_902.8157m/z			902.8157	2.27	MDAPCa2b	RC77N-E	1.43E-06
16.15_858.7656	LMGP03010100	TG(16:0/18:1/18:1)	876.8000	1.55	MDAPCa2b	RC77N-E	4.47E-06
6.31_782.5669m/z			782.5669	18.0	MDAPCa2b	RC77N-E	4.86E-06
8.77_717.5328n			717.5412	6.97	MDAPCa2b	RC77N-E	6.7E-06
6.42_807.5764n			807.5764	12.7	MDAPCa2b	RC77N-E	7.21E-06
8.13_759.5775n	LMGP01010005	PC(16:0/18:1)	760.5855	2.84	MDAPCa2b	RC77N-E	1.16E-11
16.40_912.8124n			912.8124	3.32	MDAPCa2b	RC77N-E	1.99E-5
16.41_886.7921n	LMGL03010238	TG(16:0/18:0/20:2)	886.7921	2.77	MDAPCa2b	RC77N-E	2.37 E-5
5.79_725.5567m/z			725.5567	4.12	MDAPCa2b	RC77N-E	3.85E-5
8.14_1519.1517n			1519.1517	369	MDAPCa2b	RC77N-E	5.18E-5
6.45_731.5459n	LMGP01010566	PC(16:0/16:1)	731.5459	4.77	MDAPCa2b	RC77N-E	9.13E-5
9.04_743.5509n			743.5509	2.62	MDAPCa2b	RC77N-E	1.19E-4
16.43_860.7813n			860.7813	1.57	MDAPCa2b	RC77N-E	1.2E-4
7.86_734.5698m/z	LMGP1010564	PC(16:0/16:0)	733.5622	2.67	MDAPCa2b	RC77N-E	1.39E-4
10.84_787.6085n	LMGP1010764	PC(18:0/18:1)	787.6085	2.11	MDAPCa2b	RC77N-E	1.79E-4
5.79_702.5656n	LMSP03010003	SM(d18:1/16:0)	702.5656	2.24	MDAPCa2b	RC77N-E	2.02E-4

**Table 3:** Significantly upregulated lipid molecular species amongst the five cells lines in the positive acquisition mode.

Compound LC MS/MS ID	LMID	Common Name	Exact Neutral Monoisotopic Mass	Maximum Fold increase	Highest Mean Cell Line	Lowest Mean Cell Line	P value
5.79_702.5656n	LMSP03010003	SM(d18:1/16:0)	702.5676	8.97	MDAPCa2b	RWPE-1	1.42E-9
6.16_731.5463n	LMGP01010566	PC(16:0/16:1)	731.5465	2.24	LNCAP	RC77T-E	1.05E-6
6.45_731.5459n	LMGP01010566	PC(16:0/16:1)	731.5465	31.4	LNCAP	RWPE-1	6.77 E-11
7.86_734.5698m/z	LMGP01010564	PC(16:0/16:0)	733.5622	7.01	MDAPCa2b	RWPE-1	8.69E-10
8.13_759.5775n	LMGP01010005	PC(16:0/18:1)	759.5778	8.04	MDAPCa2b	RWPE-1	7.15E-11
8.36_785.5936n	LMGP01010768	PC(18:0/18:2)	785.5935	3.73	MDAPCa2b	RWPE-1	6.07E-9
8.50_788.5450m/z	LMPG03010025	PS(18:0/18:1)	788.545	2.01	MDAPCa2b	RC77T-E	5.69E-6
10.84_787.6085n	LMGP01010754	PC(18:0/18:1)	787.6091	20.3	MDAPCa2b	RWPE-1	6.07 E-12
16.15_902.8157m/z	LMGL03010271	TG(16:0/18:1/20:2)	884.7833	3.2	LNCAP	RWPE-1	2.58E-12
16.40_912.8124	LMGL03010490	TG(18:0/18:1/20:2)	912.8146	4.23	LNCAP	RWPE-1	1.32E-11
16.41_886.7921n	LMGL03010234	TG(16:0/18:1/20:1)	886.7989	2.82	MDAPCa2b	RWPE-1	3.47E-11

**Table 4:** Significantly upregulated lipids molecular species amongst the five cells lines in the negative acquisition mode.

Compound LC MS/MS ID	LMID	Common Name	Exact Neutral Monoisotopic Mass	Maximum Fold increase	Highest Mean Cell Line	Lowest Mean Cell Line	P value
8.57_889.5800m/z	LMGP06010934	PI(18:0/20:2)	890.5884	28.8	LNCAP	RWPE-1	1.03E-11
4.90_857.5171m/z	LMGP06010958	PI(16:0/20:4)	858.5258	161	MDAPCa2b	RWPE-1	6.64E-11
4.99_883.5330m/z	LMGP06010854	PI(18:0/20:5)	884.5415	106	MDAPCa2b	RWPE-1	2.36E-11
5.76_7475651m/z	LMSP03010003	SM(d18:1/16:0)	702.5676	2.41	MDAPCa2b	RC77T-E	2.61E-6
6.19_835.5335m/z	LMGP06010001	PI(16:0/18:1)	836.5415	8.52	MDAPCa2b	LNCAP	6.45E-11
6.35_602.5604m/z	LMGP01010678	PC(16:1/18:1)	757.5622	5.46	RWPE-1	MDAPCa2b	8.47E-12
6.42_885.5489m/z	LMGP06010010	PI(18:0/20:4)	886.5571	23.2	MDAPCa2b	RWPE-1	4.95E-13
6.91_764.5233m/z	LMGP02011196	PE(18:1/20:4)	765.5309	118	MDAPCa2b	RWPE-1	4.14E-13
7.05_790.5464m/z	LMGP02010864	PE(20:1/20:5)	791.5465	133	MDAPCa2b	RWPE-1	2.76E-11
7.17_887.5643m/z	LMGP06010855	PI(18:0/20:3)	888.5728	69	LNCAP	RWPE-1	2.26E-11
7.83_887.5649m/z	LMGP06010568	PI(20:3/18:0)	888.5728	25.5	LNCAP	RWPE-1	5.31E-13
8.50_788.5450m/z	LMGP03010025	PS(18:0/18:1)	789.552	5.2	MDAPCa2b	RWPE-1	5.63E-11
8.80_716.5236m/z	LMGP02010009	PE(16:0/18:1)	717.5309	3.22	LNCAP	RWPE-1	5.63E-11
9.01_742.5396m/z	LMGP02010052	PE(18:1/18:1)	743.5465	1.62	RC77N-E	RC77T-E	5.69E-6
9.01_766.5392m/z	LMGP02010118	PE(18:0/20:4)	767.5465	383	MDAPCa2b	RWPE-1	2.64E-14
9.14_792.5548m/z	LMGP02010652	PE(18:1/22:4)	793.5622	1.50E+03	MDAPCa2b	RWPE-1	1.32E-7
10.05_700.5286m/z	LMGP02030095	PE(P-16:0/18:1)	701.5359	53.1	RC77N-E	MDAPCa2b	3.37E-11
10.81_832.6070m/z	LMGP01010761	PC(18:0/18:1)	787.6091	19.9	MDAPCa2b	RWPE-1	5.79E-12
11.64_744.5550m/z	LMGP02010036	PE(18:0/18:1)	745.5622	8.98	MDAPCa2b	RWPE-1	1.77E-11
12.90_728.5596m/z	LMGP02030004	PE(P-18:0/18:1)	729.5672	74.4	RC77N-E	MDAPCa2b	1.88E-11

In complementary experiments, the polar metabolite data from the cell lines were processed using the same strategy. S2 Fig shows representative chromatograms of the separation of various polar metabolites in the negative and positive electrospray ionization modes. We have performed comprehensive analyses using the same methodologies as described in the preceding section for lipids and the results are summarized in the supplementary data sections. The comparisons for differentially expressed metabolites between the different cell lines with different malignancy phenotypes are provided in Table 5. The results for the above described analyses are summarized below.

**Table 5:** Significantly upregulated metabolites amongst the five cells lines in the negative^1^ and positive^2^ acquisition modes.

Compound LC MS/MS ID	HMDID	Common Name	Exact Neutral Monoisotopic Mass	Maximum Fold increase	Highest Mean Cell Line	Lowest Mean Cell Line	P value
3.43_347.0453m/z^2^	HMDB00195	Inosine	268.0808	8.12	MDAPCa2b	LNCAP	2.50E-3
4.19_307.0838n^1^	HMDB00125	Glutathione	307.0838	627	RC77N-E	MDAPCa2b	4.18E-2
5.22_346.0552m/z^1^	HMDB03540	3'-AMP	347.0631	14	RC77N-E	RC77T-E	1.08E-8
5.33_323.0287m/z^1^	HMDB11641	Uridine 2'- phosphate	324.0359	20.8	MDAPCa2b	RC77T-E	3.45E-8
5.57_362.0504m/z^1^	HMDB01397	Guanosine monophosphate	363.058	27.6	MDAPCa2b	RC77T-E	3.24E-9
6.50_606.0743m/z^1^	HMDB00304	acetylgalactosamine	607.0816	30.3	MDAPCa2b	RC77T-E	3.91E-9
2.29_276.185m/z^2^	HMDB13131	Hydroxyhexanoycarnitine	276.185	275	MDAPCa2b	LNCAP	1.60e-7
2.74_161.1055^2^	HMDB00062	L-Carnitine	161.1055	3.2	MDAPCa2b	LNCAP	1.10e-3
5.30_612.1524m/z^2^	HMDB03337	Oxidized glutathione	612.1524	1.97	MDAPCa2b	LNCAP	1.55E-5

### Triacylglycerols

Three triacylglycerol (TAGs) species, LMGL03010271 (TG (16:0/18:1/20:2), LMGL03010490 TG(18:0/18:1/20:2), and LMGL03010234 TG(16:0/18:1/20:1) are significantly upregulated (>2 fold increase) in the metastatic cell lines LNCAP and MDAPCa2b compared to the normal control prostate epithelial cell lines RWPE-1, the non-malignant RC77N-E and the primary adenocarcinoma cell line RC77T-E.

### Glycerophospholipids

Twenty six (26) glycerophospholipids (GP) species are significantly upregulated (>2 fold increase) amongst the 5 cells lines. The following glycerophospholipids sub-species were identified glycerophosphocholine (PC) 8, glycerophosphoinositol (PI) 7, and glycerophosphoserine (PS) 2, glycerophosphoethanolamine (PE) 9. 23 out of the 26 glycerophospholipids are significantly upregulated (>2 fold increase) in the LNCAP and MDAPCa2b metastatic cells compared to the normal prostate epithelial cell lines RWPE-1, the non-malignant RC77N-E and the primary adenocarcinoma cell line RC77T-E. In contrast, two glycerophosphoethanolamines, PE (P-16:0/18:1) and PE(P-18:0/18:1) are significantly upregulated in the non-malignant RC77N-E cells compared to the bone metastatic MDAPCa2b cell line. In addition, the glycerophosphocholine PC(16:1/18:1) is significantly upregulated in the normal prostate epithelial cell lines RWPE-1 compared to the bone metastatic MDAPCa2b cell line. Another glycerophosphoethanolamine, PE (P-16:0/18:1), is also significantly upregulated in the non-malignant RC77N-E cells compared to the malignant primary adenocarcinoma RC77T-E that was derived from the same patient.

### Sphingolipids

One identified sphingomyelin (SM) species, SM(d18:1/16:0), is significantly upregulated in the metastatic cells compared to the normal control prostate epithelial cell lines RWPE-1, the non-malignant RC77N-E and the primary adenocarcinoma cell line RC77T-E.

### Differential expression of phosphatidylcholine pathway enzymes in the prostate cell lines

**Fig 6:**
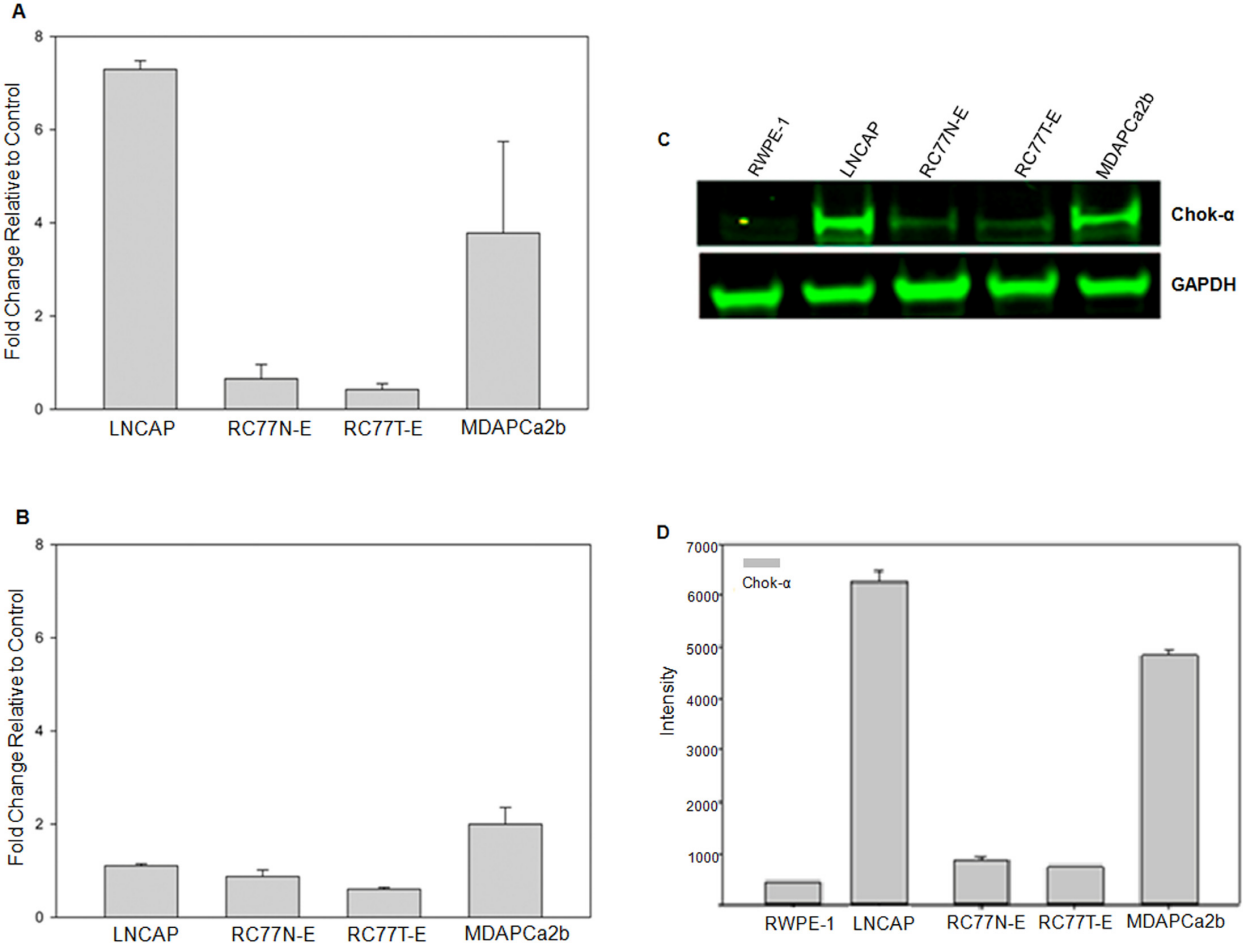
Validation of the expression profiles of *ChoK-α* and *LPCAT3* genes. (A) Real time RT-PCR analysis of expression profiles of ChoK-α and LPCAT 3. There is a significant upregulation in the expression of ChoK-α in the metastatic LNCAP and MDAPCa2b compared non-malignant RC77N-E and the malignant RC77T-E cells. (B) Western blot analysis of expression of ChoK-α and GAPDH in prostate cell lines. There is a significant upregulation in the expression of ChoK-α in the metastatic LNCAP and MDAPCa2b compared to the normal RWPE-1, non-malignant RC77N-E and the malignant RC77T-E cells. GAPDH was used as a loading control and there are no significant differences in the expression of the protein. (C) Densitometry measurements of Western Blot detection of ChoK-α and GAPDH.

Our lipidomics data demonstrates the upregulation of specific phosphatidylcholine (PC) species in aggressive prostate cancer cells. PC accounts for between 40–60% of the phospholipids of eukaryotic membranes and they play a significant role in cellular structure and multiple biological functions [19]. PC, like other phospholipids, is synthesized *de novo* in eukaryotic cells through the Kennedy pathway [20]. The Lands cycle is responsible for acyl remodeling to modify fatty acid composition of phospholipids derived from the Kennedy pathway [21]. In this process, the fatty acyl composition at the sn-2-position of PC is then altered and the net result is the generation of a diverse population of PC species that have unique fatty acids with different carbon chain lengths and with different degrees of saturation [22–24]. The enzymes that control these two pathways play a central role in the regulation of the levels of PC and other phospholipids that are available in eukaryotic cells. In turn, the levels of the phospholipids species may control biochemical processes that might significantly affect processes and pathways that lead to aggressive tumorigenic phenotype. We have performed quantitative real time qRT-PCR gene expression analyses targeting choline kinase alpha (ChoK-α) the first enzyme in the Kennedy pathway and 3 Lands cycle enzymes, lysophosphatidylcholine acyltransferases 1, 2 and 3 (LPCAT1, LPCAT2 and LPCAT3) respectively. The primers that were used for real time qRT-PCR are shown in S1 Table. There is significant over expression of ChoK-α in the metastatic MDAPCa2b and LNCaP cells compared to the other cell lines (Fig 6A). The normal RWPE-1 cells showed the lowest expression levels of ChoK-α. The expression levels of LPCAT1, LPCAT2 and LPCAT3 are similar in the five cell lines (data not shown). As an example, the fold changes in the expression of LPCAT3 in the different cells which show non-significant differences are shown in Fig 6B. The differential gene expression of ChoK-α was validated at protein level by Western blot where both the LNCaP and MDAPCa2b cells show significantly higher levels of expression of ChoK-α protein compared to their non-metastatic counterparts Fig 6C. The quantitation of the western blot showing differential expression of ChoK-α was confirmed by densitometry (Fig 6D). GAPDH was used as loading control for the Western blot. Additional, comparative transcriptomic and biochemical analysis between the LNCAP cells and its aggressive sub-clone C4-2 further confirm a significant upregulation of ChoK-α in the aggressive cells (S3 Fig). Therefore, our genomic and biochemical data provide further supporting evidence that overexpression of ChoK-α may be involved in a potential role for the upregulation of different PC species which may be involved in the development aggressive prostate cancers.

### Hydrophilic metabolites

A total of 9 identified metabolites were found to be significantly differentially expressed between the five cell lines and the results are summarized in Table 4. The most significantly differentially upregulated metabolites include glutathione, which shows the highest upregulation in the non-malignant RC77N-E cells (600 fold increase) as compared to the bone metastatic MDAPCa2b cells. Our observation is consistent with previous studies demonstrating a decrease in glutathione and an increase in cysteine levels in human prostate cancer (PCa) tissues with an increase in Gleason scores, indicating redox imbalance with PCa progression [25, 26]. In contrast, hydroxyhexanoylcarnitine is significantly upregulated (275 fold increase) in the MDAPCa2b cells compared to all the other cells including the metastatic LNCaP cells. Hydroxyhexanoycarnitine is involved in cell signaling and in the maintenance of membrane integrity and stability, processes that may influence disease progression and severity.

## Discussion and Conclusions

Recent studies have provided data demonstrating that the progression of different cancers is accompanied by increased de novo lipid biosynthesis [27]. This study provides the first comparative lipidomics/metabolomics analysis of prostate cancer cell lines with different ethnic and tumorigenic phenotypes. Our experimental objective was to apply comparative metabolomic and lipidomic analyses to profile and identify differentially expressed molecular species in prostate cancer cells with different phenotypic backgrounds. Lipids play an important role in several biological functions and cellular processes, including membrane composition and regulation, energy metabolism, and signal transduction [28]. Lipids have also been implicated in carcinogenesis [29]. In particular, lipids, such as phosphatidylcholine and fatty acids have been reported to play a central role in the development and progression of prostate cancer [30–32]. Global lipidomics analysis provides detailed information on a wide range of individual lipid metabolites that are expressed in cells and tissues. Using this approach, we have identified potentially interesting species of different lipid subclasses including phosphatidylcholines, phoshphatidylethanolamines, and glycerophosphoinositols that are differentially regulated in prostate cancer lines.

Our study, which uses cells with different tumorigenic phenotypes, shows specific differences in the phospholipid profiles between normal, primary adenocarcinoma and cells derived from distant metastases sites. The most significant fold difference between metastatic and normal cells was found in the phosphatidylethanolamine species PE(18:1/22:4) and glycerophosphoinositol species PI(16:0/20:4), which show a maximum over 1.50E+03 fold and 161 fold increase in the metastatic MDAPCa2b cells versus the normal prostate epithelial cell line RWPE-1. There is also a significant increase in the expression levels of 7 species identified phospholipids in the bone metastatic MDAPCa2b compared to the non-malignant RC77N-E and the primary adenocarcinoma RC77T-E cells. Previous studies have demonstrated altered systemic lipid metabolism in cancer patients, as well as aberrant lipid utilization by tumor cells. Abnormalities and alterations in phospholipid metabolism are therefore mostly likely to be associated with malignant transformation, tumorigenicity, metastasis and aggressive prostate cancer disease progression. Importantly, this article demonstrates a trend with an upregulation of specific sub-species of phospholipids in the metastatic prostate cancer cells.

These results suggest that alterations in expression of specific lipidomes may play an important role in the pathogenesis and progression of prostate cancer. Therefore the lipids that we have demonstrated to be significantly upregulated in the metastatic LNCAP and MDAPCa2b cells are potentially markers of aggressive metastatic prostate cancer disease. The significant upregulation of specific metabolites in the aggressive metastatic MDAPCa2b cells including hydroxyhexanoylcarnitine would suggest the existence of the differential activation of specific pathways in this cell line.

Using the information that we have gained from our lipidomics and metabolomics studies using cell lines, we will apply the same global experimental strategy using a large set of stratified patient tissue samples. Disease stratified prostate tissues obtained from prostate cancer patients will be used to determine the expression profiles of phospholipids and their potential to serve as prognostic markers for disease progression. In conclusion, our study demonstrates that comparative quantitative lipidomics and metabolomics analysis may provide insights into novel pathways, identify metabolites and lipids that will assist us to understand their role in prostate cancer progression. Some of these pathways and molecules will potentially assist in the development of novel treatment and diagnostic strategies for prostate and other cancers.

## Supporting Information

S1 Fig Low- and high-energy spectra for the identification of a differentially expressed lipid between RC77N and MDAPCa2b. (TIF)

S2 Fig Analysis of polar metabolites extracted from prostate cancer cell lines using UPLC/MS-MS. (a). A chromatogram acquired in the negative mode. (b). A chromatogram acquired in the positive mode using the same sample.
(TIF)

S3 Fig Western blot analysis of expression of ChoK-α and GAPDH in LNCaP and C4-2 prostate cell lines. There is a significant upregulation in the expression of ChoK-α in the metastatic and aggressive C4-2 cells to the less aggressive parental LNCaP. GAPDH was used as a loading control and there are no significant differences in the expression of the protein.
(TIF)

S1 Table List of primers used in RT-PCR experiments. (DOCX)
