# Supplementary figures and images for: Comparative Metabolomic and Lipidomic Analysis of Phenotype Stratified Prostate Cells
Source: PLoS One. 2015 Aug 5;10(8):e0134206. doi: 10.1371/journal.pone.0134206 (PMC4526693; doi:10.1371/journal.pone.0134206)

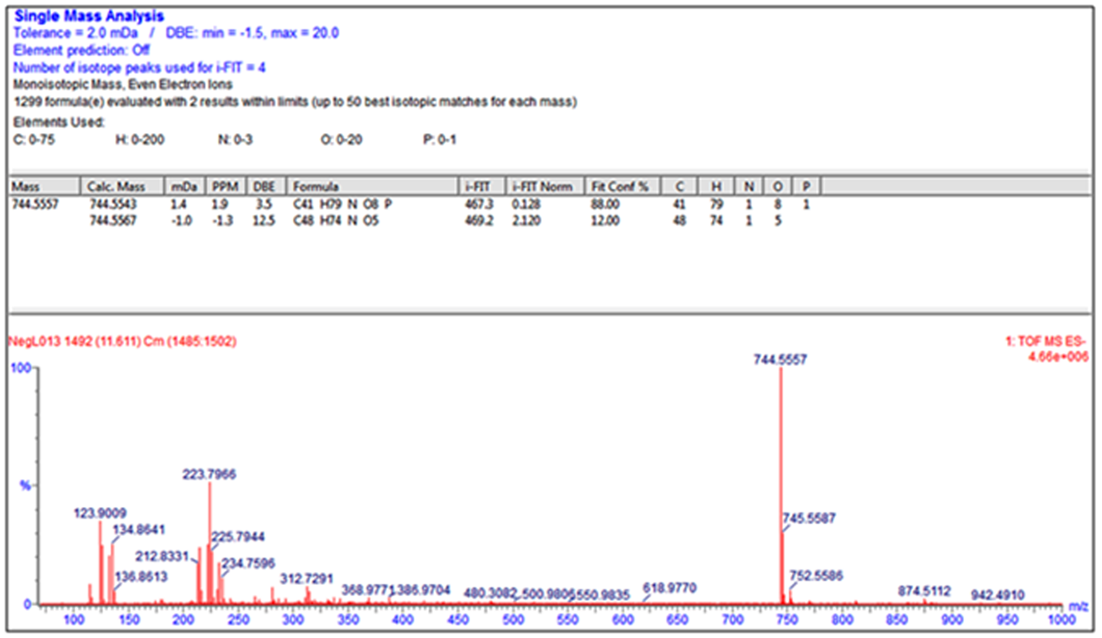

Supplement: S1 Fig — (TIF) [file pone.0134206.s001.tif]

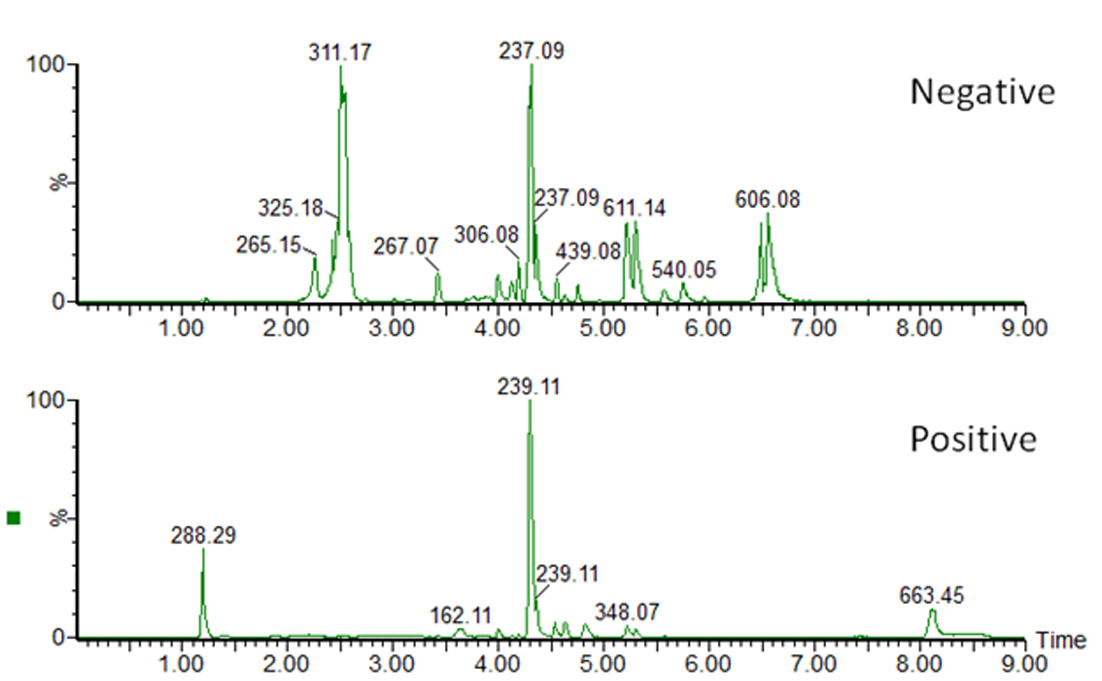

Supplement: S2 Fig — (a). A chromatogram acquired in the negative mode. (b). A chromatogram acquired in the positive mode using the same sample. (TIF) [file pone.0134206.s002.tif]

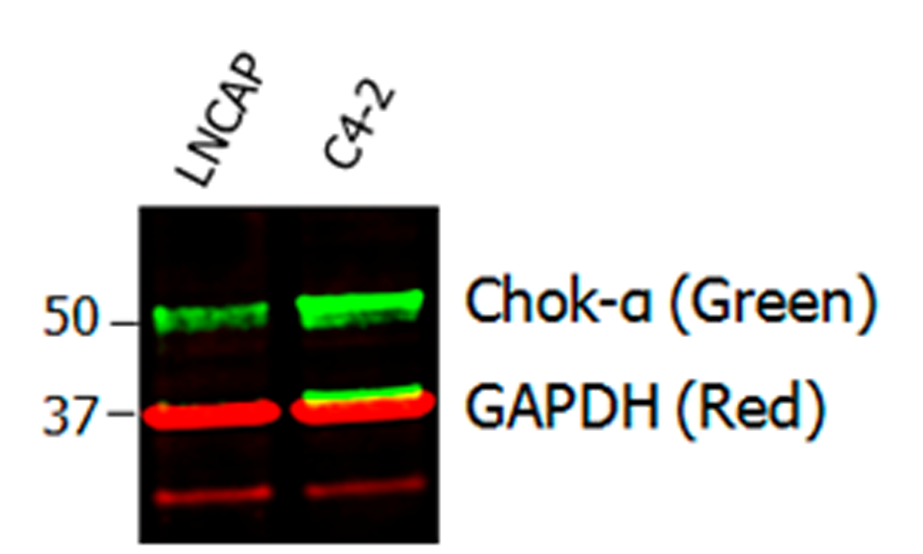

Supplement: S3 Fig — There is a significant upregulation in the expression of ChoK-α in the metastatic and aggressive C4-2 cells to the less aggressive parental LNCaP. GAPDH was used as a loading control and there are no significant differences in the expression of the protein. (TIF) [file pone.0134206.s003.tif]
