# Supplementary material for: Comparative Metabolomic and Lipidomic Analysis of Phenotype Stratified Prostate Cells
Source: PLoS One. 2015 Aug 5;10(8):e0134206. doi: 10.1371/journal.pone.0134206 (PMC4526693; doi:10.1371/journal.pone.0134206)
Supplement: S1 Table — (DOCX) [file pone.0134206.s004.docx]

**S1 Table**

| Gene | Primer |
| --- | --- |
| ChoK-α | Forward 5'-TCA GAG CAA ACA TCC GGA AGT-3' |
|  | Reverse 5'- GGC GTA GTC CAT GTA CCC AAA T-3 |
| LPCAT 1 | Forward 5'-CAT GAG GCT GCG GGG ATG -3' |
|  | Reverse 5'- TTC CCC AGA TCG GGA TGT CT-3' |
| LPCAT 2 | Forward 5'-GTC AGG CGT CCT TCT TCC C -3' |
|  | Reverse 5'- GG ACA CCA AAA CTG GTT GC-3' |
| LPCAT 3 | Forward 5'-GAA ACA AAC CCC CGC TTC AC -3' |
|  | Reverse 5'- CCT TGG CAC CAT TGC TTT GT-3' |
| GAPDH | Forward 5'-CAG CCT CAA GAT CAT CAG CA -3' |
|  | Reverse 5'- ACA GTC TTC TGG GTG GCA GT-3' |
